# Supplementary material for: Historical Review of Studies on Cyrtophorian Ciliates (Ciliophora, Cyrtophoria) from China
Source: Microorganisms. 2022 Jun 30;10(7):1325. doi: 10.3390/microorganisms10071325 (PMC9323287; doi:10.3390/microorganisms10071325)
Supplement: Supplementary file 1 [file microorganisms-10-01325-s001.zip › microorganisms-1770330-supplementary.pdf]

Supplementary Materials

Methods

Sequence selection, alignment, and phylogenetic tree construction

In total 108 cyrtophorian SSU rDNA sequences (NCBI accession numbers see Figure 5) were used to reconstruct the phylogenetic tree. Three sequences of Suctoria served as out groups. Two sequences of Chonotrichia were also added for their nestness within Dysteriida [87]. Raw sequences were aligned using the MUSCLE package on the European Bioinformatics Institute website (http://www.ebi.ac.uk) with default parameters. The resulting alignment was then trimmed at both ends, resulting in a consensus matrix of 1704 positions.

Two algorithms were employed. IQ-TREE multicore version 1.6.12 [95] was used to conduct a maximum likelihood (ML) analysis with 1000 ultrafast bootstrap replicates [96]. The model TIM2+F+R10 was chosen as the best-fit model by the in-built ModelFinder program according to Bayesian Information Criterion (BIC). A Bayesian inference (BI) analysis was run using MrBayes on XSEDE 3.2.7a package [97] on the CIPRES (http://www.phylo.org/) [98]; TPM2uf+I+G was selected as the best evolutionary substitution model using jModelTest2 on XSEDE [99] by BIC. The Markov chain Monte Carlo simulations were run for four million generations. The sample frequency was every 100th generations, with the first one milion simulations discarded as burn-in. The average standard deviation of split frequencies was 0.004110 (<0.01).
